# Anticancer potential of acetone extracts from selected *Potentilla* species against human colorectal cancer cells

**DOI:** 10.3389/fphar.2022.1027315

**Published:** 2022-09-29

**Authors:** Daniel Augustynowicz, Marta Kinga Lemieszek, Jakub Władysław Strawa, Adrian Wiater, Michał Tomczyk

**Affiliations:** ^1^ Department of Pharmacognosy, Medical University of Bialystok, Bialystok, Poland; ^2^ Department of Medical Biology, Institute of Rural Health, Lublin, Poland; ^3^ Department of Industrial and Environmental Microbiology, Maria Curie-Skłodowska University, Lublin, Poland

**Keywords:** *Potentilla*, Rosaceae, LC-PDA-HRMS, polyphenols, colorectal cancer, LS180 cells, cytotoxicity

## Abstract

Cinquefoils have been widely used in local folk medicine in Europe and Asia to manage various gastrointestinal inflammations and/or infections, certain forms of cancer, thyroid gland disorders, and wound healing. In the present paper, acetone extracts from aerial parts of selected *Potentilla* species, namely *P. alba* (PAL7), *P. argentea* (PAR7), *P. grandiflora* (PGR7), *P. norvegica* (PN7), *P. recta* (PRE7)*,* and the closely related *Drymocalis rupestris* (syn. *P. rupestris*) (PRU7), were analysed for their cytotoxicity and antiproliferative activities against human colon adenocarcinoma cell line LS180 and human colon epithelial cell line CCD841 CoN. Moreover, quantitative assessments of the total polyphenolic (TPC), total tannin (TTC), total proanthocyanidins (TPrC), total flavonoid (TFC), and total phenolic acid (TPAC) were conducted. The analysis of secondary metabolite composition was carried out by LC-PDA-HRMS. The highest TPC and TTC were found in PAR7 (339.72 and 246.92 mg gallic acid equivalents (GAE)/g extract, respectively) and PN7 (332.11 and 252.3 mg GAE/g extract, respectively). The highest TPrC, TFC, and TPAC levels were found for PAL7 (21.28 mg catechin equivalents (CAT)/g extract, 71.85 mg rutin equivalents (RE)/g extract, and 124.18 mg caffeic acid equivalents (CAE)/g extract, respectively). LC-PDA-HRMS analysis revealed the presence of 83 compounds, including brevifolincarboxylic acid, ellagic acid, pedunculagin, agrimoniin, chlorogenic acid, astragalin, and tiliroside. Moreover, the presence of tri-coumaroyl spermidine was demonstrated for the first time in the genus *Potentilla*. Results of the MTT assay revealed that all tested extracts decreased the viability of both cell lines; however, a markedly stronger effect was observed in the colon cancer cells. The highest selectivity was demonstrated by PAR7, which effectively inhibited the metabolic activity of LS180 cells (IC_50_ = 38 μg/ml), while at the same time causing the lowest unwanted effects in CCD841 CoN cells (IC_50_ = 1,134 μg/ml). BrdU assay revealed a significant decrease in DNA synthesis in both examined cell lines in response to all investigated extracts. It should be emphasized that the tested extracts had a stronger effect on colon cancer cells than normal colon cells, and the most significant antiproliferative properties were observed in the case of PAR7 (IC_50_ LS180 = 174 μg/ml) and PN7 (IC_50_ LS180 = 169 μg/ml). The results of LDH assay revealed that all tested extracts were not cytotoxic against normal colon epithelial cells, whereas in the cancer cells, all compounds significantly damaged cell membranes, and the observed effect was dose-dependent. The highest cytotoxicity was observed in LS180 cells in response to PAR7, which, in concentrations ranging from 25 to 250 μg/ml, increased LDH release by 110%–1,062%, respectively. Performed studies have revealed that all *Potentilla* species may be useful sources for anti-colorectal cancer agents; however, additional research is required to prove this definitively.

## Introduction

The modern world struggles with the increasing problem of cancer, a significant cause of death worldwide. In 2020, the third most commonly diagnosed type of cancer after breast and lung cancers was colorectal cancer, estimated to represent 10.0% of total cancer cases and the second leading cause of cancer death (9.4% of total cancer deaths) (Sung et al., 2021). However, due to the Western lifestyle, which is closely associated with low physical activity, a high-fat diet, and high red meat consumption, the projected number of global new colorectal cancer cases will rise from 1.93 million in 2020 to 3.15 million cases in 2040 (Xi and Xu, 2021). Therefore, the economic burden of treatment and the high mortality rate of patients resulting from cancer recurrence after chemotherapy suggests a significant need for more efficient and safer drug candidates. However, access to the most effective and modern diagnostic methods and treatments is limited for a large proportion of people. Especially in rural areas, people predominantly still depend on phytotherapy (Edgar et al., 2007). Notably, *Potentilla* species, known as cinquefoils, are widely used, since they are well known in traditional medicine throughout the Asian and European continents as valuable phytomedicines in a remedy *inter alia* against diarrhoea, ulcers, fever, jaundice, oral inflammations, topical infections, and thyroid gland disorders (Tomczyk and Latte, 2009). Moreover, ancient Chinese medical works, in particular *Compendium of Chinese Materia Medica* and *Mingyi Bielu* mentioned that aerial parts of two *Potentilla* species, namely *P. indica* and *Duchesnea chrysantha* were used as anticancer agents in monotherapy or as a main ingredient of complex formulas against unspecified types of cancers (Peng et al., 2009). A number of studies have reported on the abundance of secondary metabolites in *Potentilla* species, which determine their anti-inflammatory, antimicrobial, and antioxidative properties (Augustynowicz et al., 2021b). Moreover, earlier studies on several *Potentilla* species have shown their anti-cancer potential against various cell lines, e.g., triterpenoids isolated from *P. chinensis* were cytotoxicity against MCF7 (human breast cancer), Hep G2 (human hepatocellular carcinoma) and T84 (human colonic adenocarcinoma), while extracts and fractions from aerial parts of *P. alba* decreased proliferation and viability of HT-29 (human colon adenocarcinoma) (Zhang et al., 2017; Kowalik et al., 2020).

We hypothesized that aerial parts of selected *Potentilla* species, similarly to other species from this genus, would exhibit broad pharmacological potential. Therefore, the primary aim of our study was to assess their cytotoxicity and antiproliferative activities against human colon adenocarcinoma cell line LS180 and human colon epithelial cell line CCD841 CoN. Moreover, we identified the marker metabolites present in extracts through LC-PDA-HRMS analysis to uncover correlations between the qualitative chemical composition of extracts and their possible mechanism of action.

## Materials and methods

### Reagents

The reference substances, including procyanidin B1, procyanidin B2 and procyanidin C1 were obtained from Cayman Chemical (Ann Arbor, MI, United States). Quercetin 3-*O-*glucuronide, kaempferol 3-*O*-glucuronide and isorhamnetin 3-*O*-glucoside were obtained from Extrasynthese (Genay, France) (+)-Catechin, (-)-epicatechin and gallic acid were the products of Carl Roth (Karlsruhe, Germany). Quercetin 3-*O*-glucoside, quercetin 3-*O*-rutinoside, kaempferol 3-*O*-glucoside (purity >96%) were isolated from flowers of *Ficaria verna* L. Hud (Ranunculaceae) (Gudej and Tomczyk, 1999). Quercetin 3-*O*-galactoside (purity >96%) was isolated from aerial parts of *Rubus saxatilis* L. (Rosaceae) (Tomczyk and Gudej, 2005) and pedunculagin was isolated from leaves of *Rubus caesius* L. (Rosaceae) (Grochowski et al., 2020). Quercetin 3-*O*-arabinofuranoside, ellagic acid and tiliroside (purity >96%) were isolated from aerial parts of *Drymocalis rupestris* (L.) Soják (Rosaceae) (Tomczyk, 2011). Agrimoniin and ellagic acid 3,3′-di-*O*-methyl ether 4-*O*-xyloside (purity >96%) were isolated from aerial parts of *P. recta* (Tomczyk, 2011; Bazylko et al., 2013). Apigenin and 3-*O*-caffeoylquinic acid (purity >96%) were isolated from leaves and inflorescences of *Arctium tomentosum* Mill. (Asteraceae) (Strawa et al., 2020). All other chemicals of analytical grade used in the study were purchased from Sigma-Aldrich (St. Louis, MO, United States). A POLWATER DL3-100 Labopol (Kraków, Poland) assembly was used to obtain ultra-pure water. Stock solutions of investigated extracts (100 mg/ml), as well as 5-fluorouracil (50 mM), were prepared by dissolving the compounds in dimethyl sulfoxide (DMSO) (POCH, Gliwice, Poland). Working solutions of investigated compounds were prepared by dissolving an appropriate stock solution in a culture medium. The final concentration of DMSO in all working solutions used in the studies was the same including control and it was 0.25%.

### Plant materials and procedure of plant extracts preparation

Seeds of five species, namely *P. alba* (ind. sem. 354), *P. grandiflora* (ind. sem. 758), *P. norvegica* (ind. sem. 303), *P. recta* (ind. sem. 1549) and *P. rupestris* (ind. sem. 763) were kindly provided by the Botanical Garden of Vilnius University (Vilnius, Lithuania), Giardino Botanico Alpino (Cogne, Italy), Hortus Botanicus Universitatis Masarykianae (Brno, Czech Republic) and Hortus Botanicus University of Tartu (Tartu, Estonia). Plants were cultivated in common plots at the Medicinal Plant Garden at the Medical University of Białystok (Białystok, Poland), and aboveground materials were collected in June-August 2016–2019. Aerial parts of *P. argentea* were collected in June-July 2017–2019 from natural habitat, at Puszcza Knyszyńska (Poland, 53°15′6″N 23°27′58″E). The taxonomic identification of plant material was carefully authenticated by one of the authors (M.T.). Voucher specimens of *P. alba* (PAL-17039), *P. argentea* (PAR-02009), *P. grandiflora* (PGR-06020), *P. norvegica* (PNO-08024), *P*. *recta* (PRE-06019) and *P. rupestris* (PRU-06021) have been deposited at the Herbarium of the Department of Pharmacognosy, Medical University of Białystok (Poland). Collected dried materials were subsequently finely grounded with an electric grinder and stored in air-tight containers at ambient temperature. Powdered dry plant materials (2.0 g each time) were separately submitted to ultrasound-assisted extraction with 70% acetone (3 × 50 ml) using an ultrasonic bath (Sonic-5, Polsonic, Warszawa, Poland) at a controlled temperature (40 ± 2 °C) for 45 min in a 1:75 (*w:v*) solvent ratio. The obtained raw extracts after solvent evaporation were diluted with water (50 ml) and subsequently portioned with chloroform (10 × 20 ml). The acetone extracts were obtained using this method for *P. alba* (**PAL7**), *P. argentea* (**PAR7**), *P. grandiflora* (**PGR7**), *P. norvegica* (**PN7**), *P. recta* (**PRE7**) and *P. rupestris* (**PRU7**).

### Determination of total phenolic content

The total phenolic content (TPC) was measured by the Folin-Ciocalteu assay with some modifications (Slinkard and Singleton, 1977). Briefly, 25 µl of tested solution (1 mg/ml) was mixed with 100 µl of diluted Folin-Ciocalteu reagent (1:9, *v*/*v*) and the mixture was allowed to react for 3 min. Thereafter, 75 µl of 1% Na_2_CO_3_ solution was added and the prepared mixture was incubated for 2 h at ambient temperature. The absorbance was measured at 760 nm using a microplate reader EPOCH2 BioTech (Winooski, VT, United States). The TPC determination was repeated at least three times for each sample solution. Obtained results were expressed as milligrams of gallic acid equivalents per Gram of dry extract (mg GAE/g extract).

### Determination of total tannin content

The total tannin content (TTC) of each extract was measured by the employment of the protein-binding method and Folin-Ciocalteu assay described in the European Pharmacopoeia 10th ed (European Pharmacopoeia, 2019). with modifications. Briefly, each extract dissolved in water (1 mg/ml) was partitioned into two parts. For the first part of extracts total polyphenols were determined for each aliquot (25 µl) by mixing with 100 µl of diluted Folin-Ciocalteu reagent (1:9, *v/v*). After 3 min 75 µl of 1% Na_2_CO_3_ was added and the mixture was allowed to stand for 2 h at room temperature. Thereafter the absorbance of each sample (A_1_) was recorded at 760 nm using a EPOCH2 microplate reader. Subsequently, the second part of aliquots of 0.5 ml each were mixed with 10 mg of hide powder. These preparations were shaken for 1 h without light and then centrifugated. A 25 µl of supernatants were assayed for total polyphenolics as described above and the absorbance of each sample (A_2_) was recorded at 760 nm. Afterwards, the total tannin content was determined by subtraction of absorbances of total polyphenols (A_1_) from total non-tannin polyphenols (A_2_) and the obtained absorbance values were referred to a gallic acid calibration curve to obtain their values as milligrams of gallic acid equivalents per Gram of dry extract (mg GAE/g extract). The determination of TTC was repeated at least three times for each sample solution.

### Determination of total proanthocyanidin content

The total proanthocyanidin content (TPrC) was analysed with the employment of a 4-dimethylamino-cinnamaldehyde (DMCA) reagent (Feliciano et al., 2012). The analysis was carried out in a microplate reader. A 50 µl of sample solution (1 mg/ml) dissolved in methanol was mixed with 250 µl of 0.1% DMCA in 6 M HCl in methanol. The mixture was incubated at ambient temperature for 15 min, and thereafter, the absorbance was recorded at 635 nm. The TPrC determination was repeated at least five times for each sample solution and was expressed as milligrams of catechin equivalents per Gram of dry extract (mg CE/g extract).

### Determination of total flavonoid content

The total flavonoid content (TFC) of each extract was determined using the previously described aluminium chloride (AlCl_3_) colorimetric method (Augustynowicz et al., 2021a) with slight modifications. In brief, 100 µl of tested solution or 100 µl of blank sample (methanol) was mixed with 100 µl of 2% (*w:v*) AlCl_3_ solution. The mixture was kept at ambient temperature for 10 min. Then the absorbance of the mixture was recorded at 415 nm using a EPOCH2 microplate reader. The TFC determination was repeated at least three times for each sample solution. TFC was expressed as milligrams of rutin equivalents per Gram of dry extract (mg RE/g extract).

### Determination of total phenolic acid content

The total phenolic acid content (TPAC) determination was carried out using the procedure with the use of Arnov’s reagent (1 g of sodium molybdate and 1 g of sodium nitrate dissolved in 10 ml of distilled water) (Polumackanycz et al., 2019). A 30 µl of the tested solution, 180 µl of water, 30 µl of 0.5 M HCl, 30 µl of Arnov’s reagent and 30 µ of 1 M NaOH were sequentially added to the microplate well. After incubation of mixture at room temperature for 20 min, the absorbance was measured at 490 nm. The TPAC determination was repeated at least three times for each sample solution and the obtained values were expressed as milligrams of caffeic acid equivalents per Gram of dry extract (mg CAE/g extract).

### Estimation of qualitative composition with the employment of LC-PDA-HRMS

Evaluation of the secondary metabolite composition of each extract was conducted using an Agilent 1260 Infinity LC chromatography system coupled to a photo-diode array (PDA) detector and 6230 time-of-flight (TOF) mass spectrometer (Santa Clara, CA, California). The MS conditions were as follows: electrospray ionization (ESI) source in both negative and positive ionization mode, drying and sheath gas flow 11 L/min and temperature of 350°C, nebulizer pressure of 60 psi, capillary voltages of 2,500 and 4500 V for negative and positive ion modes, respectively and fragmentor experiments at 60, 180 and 320 V. The data were collected in the 120–3,000 m*/z* range. The separation was performed using a Kinetex XB-C18 column (150 × 2.1 mm, 1.7 µm, Phenomenex, Torrance, CA, United States). The mobile phases were ultra pure water (A) and acetonitrile (B) with 0.2% formic acid. The separation was achieved by a gradient of 0–3 min 65% B; 3–35 min 1% B, 35–80 min 12% B, 80–113 min 45% B, extended by 7 min of equilibrating time. The flow rate was 0.2 ml/min, and the column temperature was maintained at 35 ± 0.8°C. The UV-vis spectra were recorded in the range of 190–540 nm with selective wavelength monitoring at 280 and 360 nm. Data were processed with the employment of MassHunter Qualitative 10.0. Analysis software. Compounds were characterized based on UV–Vis and MS spectra and retention time of standards.

### Cell cultures

Human colonic epithelial cell line CCD841 CoN was purchased from the American Type Culture Collection (ATCC, Manassas, VA, United States). Human colon adenocarcinoma cell line LS180 was obtained from the European Collection of Cell Cultures (ECACC, Centre for Applied Microbiology and Research, Salisbury, United Kingdom). Cell cultures were conducted in accordance with the guidelines of the collections in which they were purchased.

### Examination of the anticancer potential of extracts

Both colon epithelial, as well as colon adenocarcinoma cells, were seeded on 96-well microplates at a density of 5 × 10^4^ cells/mL. The following day, the culture medium was exchanged for fresh medium supplemented with investigated extracts or 25 μM 5-fluorouracil (5-FU). After 48 h of cell treatment, the compounds’ antiproliferative effect was determined using Cell Proliferation ELISA BrdU, following the manufacturer’s instructions (Roche Diagnostics GmbH, Penzberg, Germany), while the compounds’ cytotoxicity was examined by the *In Vitro* Toxicology Assay Kit Lactate Dehydrogenase Based according to the manufacturer’s instructions (Sigma-Aldrich, St. Louis, MO, United States). Furthermore, cell viability in response to 48 h of exposure to investigated compounds was determined by MTT assays. A detailed description of the execution of the above-mentioned assays was presented by Langner and co-authors (Langner et al., 2019).

### Statistical analysis

The analysed data were presented as the mean ± SEM. Statistical analyses were performed using One way-ANOVA with the Tukey *post-hoc* test and column statistics. Statistical significance was accepted at *p* < 0.05. The IC_50_ value (concentration leading the 50% inhibition of proliferation compared to the control) was calculated using GraphPad PRISM.

## Results

In the first set of experiments, the studied extracts were examined for their TPC, TTC, TPrC, TFC, and TPAC using colorimetric methods. The obtained results are presented in Table 1. **PAR7** and **PN7**, followed by **PRU7**, were found to contain the highest TPC (339.72, 332.11, and 304.08 mg GAE/g extract, respectively) and TTC (246.92, 252.30, and 209.43 mg GAE/g extract, respectively). **PAL7** had the lowest TPC and TTC values (159.87 and 84.89 mg GAE/g). However, **PAL7** was found to contain the highest TPrC (21.28 mg CE/g extract), while the other extracts had low proanthocyanidin content. The TFC levels for all tested samples were found to be similar, with the highest values for **PAL7** and **PAR7** (71.85 and 56.79 mg RE/g extract, respectively). Moreover, the highest TPAC values were revealed for **PAL7** and **PN7** (124.18 and 78.95 mg CAE/g extract, respectively).

**TABLE 1:** Total phenolic (TPC), tannin (TTC), proanthocyanidin (TPrC), flavonoid (TFC) and phenolic acid contents (TPAC) of selected acetone extracts of *Potentilla* species.

Samples	TPC (mg GAE/g extract)	TTC (mg GAE/g extract)	TPrC (mg CE/g extract)	TFC (mg RE/g extract)	TPAC (mg CAE/g extract)
**PAL7**	159.87 ± 1.79	84.89 ± 1.40	21.28 ± 0.04	71.85 ± 1.40	124.18 ± 1.18
**PAR7**	339.72 ± 5.29	246.92 ± 4.64	6.95 ± 0.07	56.79 ± 0.98	78.95 ± 0.90
**PGR7**	228.36 ± 3.40	156.53 ± 3.71	3.80 ± 0.06	47.61 ± 0.35	58.61 ± 0.34
**PN7**	332.11 ± 1.40	252.30 ± 1.70	1.14 ± 0.02	38.06 ± 0.79	92.78 ± 1.03
**PRE7**	257.68 ± 2.95	170.45 ± 2.86	2.70 ± 0.08	43.37 ± 0.84	75.20 ± 1.23
**PRU7**	304.08 ± 2.51	209.43 ± 2.57	1.11 ± 0.02	47.74 ± 0.73	55.45 ± 0.59

^*^GAE, gallic acid equivalent; CE, catechin equivalent; RE, rutin equivalent; CAE, caffeic acid equivalent.

To unveil the secondary metabolite composition, acetone extracts of selected *Potentilla* species were analysed via LC-PDA-HRMS. The analysis demonstrated the presence of 83 compounds, predominately polyphenolic compounds, ascribed to hydrolysable and condensed tannins, flavonoids, and phenolic acids. Hydrolysable tannins were present in all extracts with the exception of **PAL7**, primarily represented by ellagitannins, such as pedunculagin α and β (**3**,**8**), agrimoniin (**69**), laevigatin isomers (**39**, **40**, **47**), galloyl-HHDP-glucose isomers (**6**, **9**, **18**), galloyl-bis-HHDP-glucose isomers (**38**, **42**, **63**), ellagic acid (**51**), ellagic acid 3,3′-di-*O*-methyl ether 4-*O*-xyloside (**75**), ellagic acid 3′-*O*-methyl ether 4-*O*-xyloside (**54**), and ellagic acid 3′-*O*-methyl ether 4-*O*-arabinoside (**70**). **PRU7** showed the presence of gallotannins, such as tri-, tetra-, and pentagalloylglucose isomers (**32**, **55**, **72**). Moreover, the degradation products of hydrolysable tannins, namely brevifolincarboxylic acid **19**) and brevifolin (**33**), were found. On the other hand, **PAL7** and **PAR7** were rich in the condensed tannins, such as (+)-catechin **13**) and (-)-epicatechin (**27**), and oligomeric procyanidins, such as procyanidin B1 (**12**), B2 (**20**), and C1 (**37**). A number of flavonoids were detected and characterized, including apigenin **78**) and its *O*-hexoside (**68**), isorhamnetin (**36**, **64**, **71**, **73**, **74**), kaempferol (**31**, **59**, **61**, **62**, **66**, **67**, **77**, **79–81**), and quercetin (**23**, **24**, **26**, **41**, **43–48**, **52**, **56–58**, **60**, **65**, **76**) derivatives. In addition, phenolic acids were also present in extracts, such as gallic acid (**1**), caffeic acid derivatives (**5**, **14**, **17**, **28**, **30**), *p*-coumaroylquinic acid isomers (**10**, **11**, **25**, **29**), and N1, N5, N10-tricoumaroyl spermidine (**83**). The detailed chromatographic data are shown in Table 2 and in Supplementary Figures S1–S6.

**TABLE 2:** MS and UV-Vis data of compounds detected in acetone extracts prepared from aerial parts of selected *Potentilla* species.

No.	Compounds	Rt [min]	UV spectra [λ max nm]	Observed^a^	Δ [ppm]	Formula	Fragmentation		Presence in extracts						Ref
							Negative	Positive	PAL7	PAR7	PGR7	PN7	PRE7	PRU7	
**1**	Gallic acid	5.70	270	169.01370	-3.44	C_7_H_6_O_5_	**169**, 125			+	+	+	+	+	(s)
**2**	2-Pyrone-4,6-dicarboxylic acid	6.65	316	182.99276	-3.46	C_7_H_4_O_6_	366, **182**, 139			+	+	+	+	+	Wilkes and Glasl, (2001)
**3**	Pedunculagin α or β	15.35	260sh	783.06883	0.38	C_34_H_24_O_22_	**783**, 481, 301			+	+	+	+	+	Grochowski et al. (2020), (s)
**4**	Polyphenol derivative	15.70	280	337.11359	-1.03	C_13_H_22_O_10_	**193**, 125		+						
**5**	5-*O*-Caffeoylquinic acid	20.07	295sh, 325	353.08747	-0.96	C_16_H_18_O_9_	**353,** 191, 179	355, **163**	+		+	+			(s)
**6**	Galloyl-HHDP-glucose	22.26	250sh	633.07245	-0.24	C_27_H_22_O_18_	**633**, 301							+	
**7**	Unknown	22.45	276	345.11788	-2.82	C_15_H_22_O_9_	**345**, 299, 161							+	
**8**	Pedunculagin α or β	23.30	260sh	783.06929	0.85	C_34_H_24_O_22_	**783**, 481, 301			+	+	+	+	+	Grochowski et al. (2020), (s)
**9**	Galloyl-HHDP-glucose	24.07	280sh	633.07359	0.36	C_27_H_22_O_18_	**633**, 481, 301				+	+	+		
**10**	*p*-Coumaroylquinic acid isomer	24.41	308	337.09247	-0.92	C_16_H_18_O_8_	**337**, 191, 163	339, **147**	+						
**11**	*p*-Coumaroylquinic acid isomer	25.27	312	337.09218	-1.59	C_16_H_18_O_8_	**337**, 191, 163	339, **147**	+						
**12**	Procyanidin B1	26.20	280	577.13507	0.81	C_30_H_26_O_12_	**577**, 289	**579**, 291, 139		+					(s)
**13**	Catechin	27.10	280	289.07136	-1.42	C_15_H_14_O_6_	**289**, 245	**291**, 139		+			+		(s)
**14**	3-*O*-Caffeoylquinic acid	28.21	295sh, 325	353.08729	-1.45	C_16_H_18_O_9_	**353**, 191	355, **163**	+		+	+	+		Strawa et al. (2020), (s)
**15**	Digalloyl-HHDP-glucose	28.56	275	785.08369	-0.63	C_34_H_26_O_22_	785**, 301**							+	
**16**	Feruloylquinic acid isomer	29.86	295sh, 325	367.10365	0.61	C_17_H_20_O_9_	**367**, 193	369, **177**	+		+				
**17**	Caffeoylquinic acid isomer	30.91	295sh, 325	353.08779	-0.08	C_16_H_18_O_9_	**353**, 191, 179	355, **163**	+						
**18**	Galloyl-HHDP-glucose	31.61	275	633.07366	-0.27	C_27_H_22_O_18_	**633**, 463, 301			+	+	+	+	+	
**19**	Brevifolincarboxylic acid	32.11	278, 360	291.01385	-2.02	C_13_H_8_O_8_	**291**, 247	293		+	+	+	+	+	Luo et al. (2020)
**20**	Procyanidin B2	33.73	278	577.13556	0.82	C_30_H_26_O_12_	**577**, 289	**579**, 291, 139	+						(s)
**21**	Ellagic acid derivative	33.89	285sh	898.13313	-1.54	C_29_H_39_O_32_	898, 633, **301**				+				
**22**	Ellagic acid derivative	34.13	325sh	632.06474	-1.85	C_27_H_21_O_18_	632, 463, **301**				+				
**23**	Quercetin *O*-hexoso *O-*uronic acid derivative	34.20	255, 350	639.12125	1.20	C_27_H_28_O_18_	**639**, 300	**641**, 479, 303			+				
**24**	Quercetin *O*-di-uronic acid derivative	34.40	255, 355	653.09958	-0.45	C_27_H_26_O_19_	**653**, 477, 301	**655**, 479, 303		+	+		+		
**25**	*p*-Coumaroylquinic acid isomer	35.13	312	337.09285	0.02	C_16_H_18_O_8_	**337**, 191	**339**, 147	+		+				
**26**	Quercetin *O*-hexoso *O-*uronic acid derivative	35.35	270, 350	639.12105	-0.61	C_27_H_28_O_18_	**639**, 463, 301	**641**, 465, 303		+				+	
**27**	Epicatechin	35.74	280	289.07133	-1.43	C_15_H_14_O_6_	**289**, 245	**291**, 139	+						(s)
**28**	2-Caffeoylisocitric acid	36.30	300sh, 328	353.05046	-2.69	C_15_H_14_O_10_	**353**, 191, 155					+	+	+	Manyelo et al. (2020)
**29**	*p*-coumaroylquinic acid isomer	36.52	312	337.09314	-0.26	C_16_H_18_O_8_	**337**, 163	**339**, 147	+						
**30**	Caffeoylmalic acid	38.51	295sh, 326	295.04541	-1.72	C_13_H_12_O_8_	591, **295**, 179, 133	**135**	+		+	+	+		Szajwaj et al. (2011)
**31**	Kaempferol *O*-di-uronic acid derivative	38.97	265, 350	637.10470	-0.62	C_27_H_26_O_18_	637, 461, 285	**639**, 463, 287		+			+		
**32**	Trigalloylglucose isomer	39.10	276	635.08918	0.20	C_27_H_24_O_18_	**635**, 465, 313, 169							+	Luo et al. (2020)
**33**	Brevifolin	39.4	275, 350	247.02433	-1.83	C_12_H_8_O_6_	**247**, 191	**249**		+	+	+	+	+	Luo et al. (2020)
**34**	Procyanidin A-type trimer	40.51	280	863.18353	1.19	C_45_H_36_O_18_	**863**, 573, 289	**865**, 287	+						
**35**	Ellagic acid *O*-hexoside derivative	41.20	252, 365	463.05162	-0.79	C_20_H_16_O_13_	**463**, 301					+		+	
**36**	Isorhamnetin *O*-di-uronic acid derivative	41.78	254, 352	667.11555	1.29	C_28_H_28_O_19_	**667,** 315, 300	**669**, 493, 317		+	+		+		
**37**	Procyanidin C1	41.88	280	865.19924	0.68	C_45_H_38_O_18_	**865**, 577, 289	**867**, 579, 291	+						(s)
**38**	Galloyl-bis-HHDP-glucose	43.98	255	935.07947	0.30	C_41_H_28_O_26_	**935**, 633, 467, 301			+		+	+	+	
**39**	Laevigatin isomer	44.46	255	1,567.14331	-1.15	C_68_H_48_O_44_	1,567, **783**, 301			+	+	+	+	+	Fecka et al. (2015)
**40**	Laevigatin isomer	45.94	255	1,567.14331	-1.15	C_68_H_48_O_44_	1,567, **783**, 301				+		+		Fecka et al. (2015)
**41**	Quercetin *O-*hexoso *O*-deoxyhexoside isomer	46.98	255, 352	609.14615	-0.73	C_27_H_30_O_16_	**609**, 446, 299	**611**, 499, 303				+			
**42**	Galloyl-bis-HHDP-glucose	47.33	276sh	935.07900	-0.79	C_41_H_28_O_26_	935, 783, 633, 467, **301**			+		+	+	+	
**43**	Quercetin *O*-hexoso-deoxyhexo-pentoside isomer	47.90	255, 355	741.18912	-0.64	C_32_H_38_O_20_	741, 447, **300**	743, 611, 465, **303**	+						
**44**	Quercetin *O-*deoxyhexoso-*O*-hexoso-deoxyhexoside isomer	48.66	256, 356	755.20326	-0.67	C_33_H_40_O_20_	**755**, 609, 446, 299	**757**, 611, 449, 303				+			
**45**	Quercetin *O*-hexoso-pentoside isomer	49.55	255, 355	595.13019	-0.10	C_26_H_28_O_16_	595, **300**, 271	597, 465, **303**	+				+		
**46**	Quercetin *O*-hexoso-pentoside isomer	50.48	255, 355	595.13046	-0.72	C_26_H_28_O_16_	595, **300**, 271	597, 465, **303**	+					+	
**47**	Laevigatin isomer	51.09	255	1,567.14239	-1.74	C_68_H_48_O_44_	1,567, **783**, 301			+		+	+	+	Fecka et al. (2015)
**48**	Quercetin *O*-pentoso-*O*-uronic acid derivative	52.30	255, 354	609.11075	-1.11	C_26_H_26_O_17_	609, **301**	611, **479**, 303		+				+	
**49**	Ellagic acid 3′-*O*-methyl ether *O*-uronic acid derivative	54.10	254, 360	491.04709	0.29	C_21_H_16_O_14_	**491**, 315, 301			+	+	+	+		
**50**	Ellagic acid *O*-pentoside	55.7	252, 360	433.04108	0.30	C_19_H_14_O_12_	**433**, 301			+			+		
**51**	Ellagic acid	56.71	254, 370	300.99841	-1.60	C_14_H_6_O_8_	**301**, 271	**303**		+	+	+	+	+	Tomczyk (2011), (s)
**52**	Quercetin 3-*O*-glucoside	59.30	255, 355	463.08816	0.13	C_21_H_20_O_12_	**463**, 300, 271	**465**, 303	+		+	+	+		(s)
**53**	Unknown	59.80	290	435.09238	-2.77	C_20_H_20_O_11_	871, **435**, 285, 151							+	
**54**	Ellagic acid 3′- *O*-methyl ether 4-*O*-pentoside	60.40	252, 362	447.05600	-1.85	C_20_H_16_O_12_	**447**, 301							+	
**55**	Tetragalloylglucose isomer	62	278	787.09898	-1.57	C_34_H_28_O_22_	**787**, 617, 465, 169							+	Luo et al. (2020)
**56**	Quercetin 3-*O*-rutoside	63.38	256, 354	609.14571	-0.32	C_27_H_30_O_16_	**609**, 300, 271	**611**, 465, 303			+	+	+		Gudej and Tomczyk, (1999), (s)
**57**	Quercetin 3-*O*-galactoside	64.03	255, 355	463.08816	-0.70	C_21_H_20_O_12_	**463**, 300, 271	**465**, 303	+	+	+		+	+	Tomczyk and Gudej, (2005), (s)
**58**	Quercetin *O*-glucuronide	64.83	255, 355	477.06649	-1.73	C_21_H_20_O_13_	**477**, 300, 271	**479**, 303		+			+	+	(s)
**59**	Kaempferol *O*-hexoso-pentoside	64.85	265, 350	579.13594	1.19	C_26_H_28_O_15_	**579**, 284	581, 449, **287**	+						
**60**	Quercetin *O*-uronic acid derivative	66.18	256, 354	477.06713	-0.49	C_21_H_18_O_13_	**477**, 301	**479**, 303			+				
**61**	Kaempferol *O*-hexoso-pentoside	66.85	265, 350	579.13520	-0.41	C_26_H_28_O_15_	577, **284**	581, **449**, 287	+						
**62**	Kaempferol *O*-hexoside	67.40	252, 350	447.09365	-0.44	C_21_H_20_O_11_	**447**, 284	**449**, 287			+		+		
**63**	Galloyl-bis-HHDP-glucose	69	260sh	935.07978	-0.18	C_41_H_28_O_26_	**935**, 467, 301			+	+	+	+		
**64**	Isorhamnetin *O*-hexoso-pentoside	73.99	255, 352	609.14611	0.20	C_27_H_30_O_16_	**609**, 314, 271	611, 479, **317**	+				+		
**65**	Quercetin 3-*O*-arabinofuranoside	85.20	254sh, 350	433.07665	-2.12	C_20_H_18_O_11_	**433**, 300	435, **303**						+	Tomczyk (2011), (s)
**66**	Kaempferol 3-*O*-glucoside	88.33	265, 350	447.09298	-1.70	C_21_H_20_O_11_	**447**, 284	**449**, 287	+	+	+	+	+	+	Gudej and Tomczyk, (1999), (s)
**67**	Kaempferol 3-*O*-glucuronide	89.30	265, 346	461.07171	-0.92	C_21_H_18_O_12_	**461**, 285	**463**, 287		+			+	+	(s)
**68**	Apigenin *O*-hexoside	90.04	266, 340	431.09754	-0.71	C_21_H_20_O_10_	**431**, 268	**433**, 271			+				
**69**	Agrimoniin	90.30	250sh	1870.15689	-0.95	C_82_H_54_O_52_	1870, 1,085, **934**, 783, 301			+	+	+	+	+	Bazylko et al. (2013), (s)
**70**	Ellagic acid 3′-*O*-methyl ether 4-*O*-pentoside	90.42	280sh, 365	447.05604	-0.72	C_20_H_16_O_12_	**447**, 315, 301			+		+	+		Luo et al. (2020)
**71**	Isorhamnetin 3-*O*-glucoside	91.43	265, 355	477.10387	0.49	C_22_H_22_O_12_	**477**, 314	**479**, 317	+						(s)
**72**	Pentagalloyloglucose isomer	91.68	280	939.11105	0.49	C_41_H_32_O_26_	**939**, 769, 469, 169							+	
**73**	Isorhamnetin *O*-deoxyhexoso-hexoso-*O-*pentoside isomer	91.92	254sh, 355	753.18766	-0.45	C_33_H_38_O_20_	**753**, 314, 299	755, 623, **317**					+		
**74**	Isorhamnetin *O*-uronic acid derivative	92.81	255, 354	491.08356	0.77	C_22_H_20_O_13_	**491**, 315, 300	**493**, 317		+			+		
**75**	Ellagic acid 3,3′-di-*O*-methyl ether 4-*O*-xyloside	94.14	245, 370	461.07148	-1.26	C_21_H_18_O_12_	**461**, 328, 297	**463**, 331				+	+		Tomczyk (2011), (s)
**76**	Quercetin *O*-uronic acid derivative	94.80	270sh, 370	477.06758	0.10	C_21_H_18_O_13_	**477**, 301	**479**, 303				+			
**77**	Kaempferol derivative	94.90	266sh, 348	533.09391	0.27	C_24_H_22_O_14_	**533**, 489, 284	**535**, 287			+				
**78**	Apigenin	98.04	268, 338	269.04538	-2.04	C_15_H_10_O_5_	**269**, 227	**271**			+				Strawa et al. (2020), (s)
**79**	*trans*-Tiliroside	101.48	268, 315	593.12979	-0.55	C_30_H_26_O_13_	**593**, 284	**595**, 287	+	+	+	+	+	+	Tomczyk (2011), (s)
**80**	Kaempferol derivative	101.87	268, 330	623.14131	1.02	C_31_H_28_O_14_	**623**, 284	**625**, 595, 287	+				+	+	
**81**	*cis*-Tiliroside	102.37	268, 315	593.12928	0.44	C_30_H_26_O_13_	**593**, 284	**595**, 287		+	+	+	+	+	Luo et al. (2020)
**82**	Unknown	102.54	280	445.18621	-1.13	C_24_H_30_O_8_	**445**, 385		+					+	
**83**	N^1^, N^5^, N^10^-tricoumaroyl spermidine	104.47	295, 310sh	582.26072	-0.06	C_34_H_37_N_3_O_6_	**582**, 462, 342, 285	**584**, 438, 292, 147	+	+	+	+	+	+	Elejalde-Palmett et al. (2015)

a Exact mass of [M-H]^-^ ion; sh–peak shoulder; bold–most aboundantion; (s)—reference substance; HHDP, hexahydroxydiphenoyl group.

**FIGURE 1:**
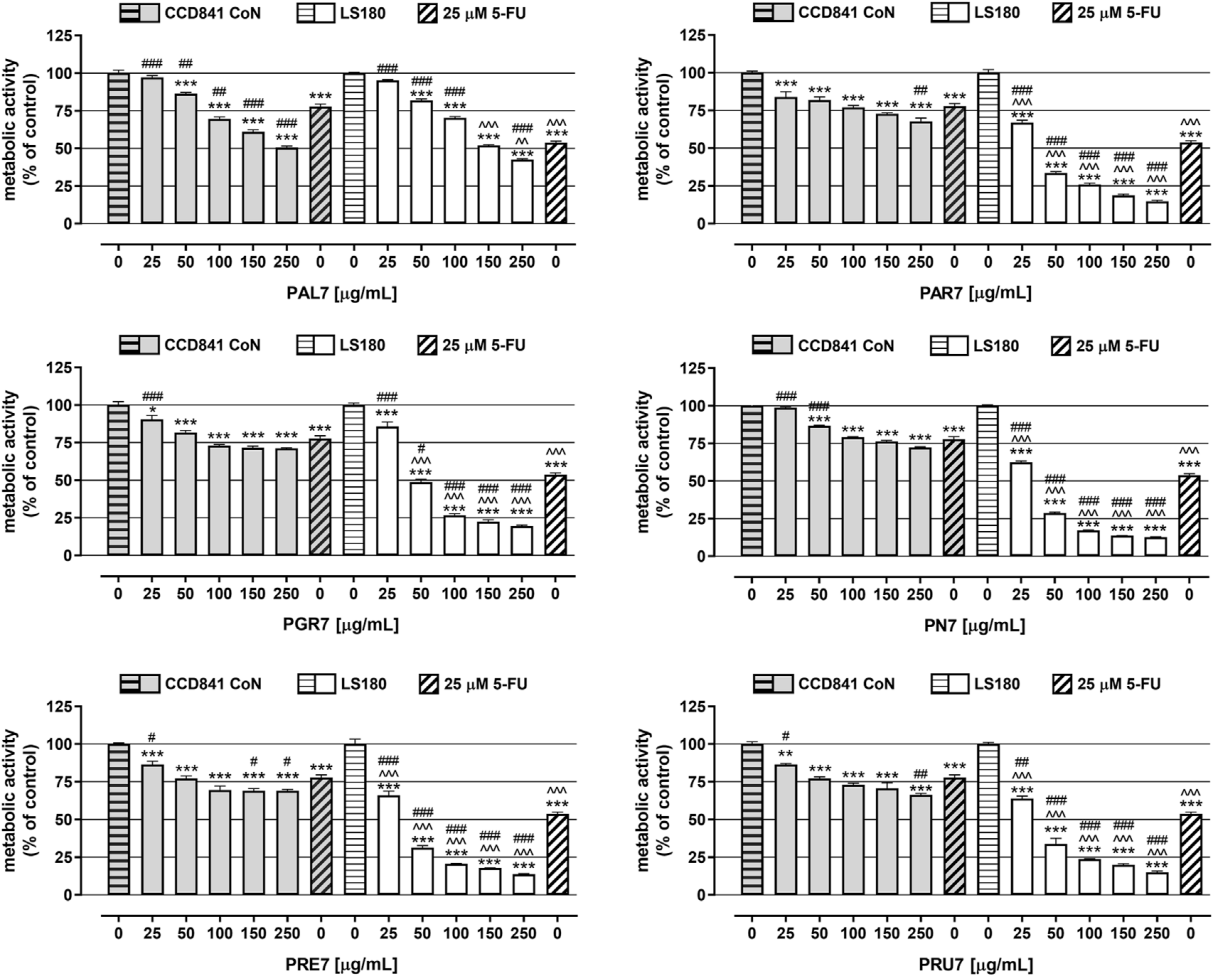
Influence of acetone extracts isolated from the aerial parts of *Potentilla* L. on the viability of human colon epithelial cell line CCD841 CoN and human colon adenocarcinoma cell line LS180. The cells were exposed for 48 h to the culture medium alone (control), or extract at concentrations of 25, 50, 100, 150, and 250 μg/ml, or 25 μM 5-fluorouracil (5-FU; positive control). The metabolic activity of investigated cell lines in response to tested compounds was examined photometrically by means of MTT assay. Results are presented as mean ± SEM of at least six measurements. *p < 0.05; **p < 0.01; ***p < 0.001 vs. control, #*p* < 0.05; ##*p* < 0.01; ###*p* < 0.001 vs. positive control, ^^*p* < 0.01; ^^^*p* < 0.001 colon cancer cells treated with extract/5-FU vs. colon epithelial cells exposed to the extract/5-FU at the corresponding concentration; one-way ANOVA test; post-test: Tukey.

In the next set of experiments, the viability of both human colon epithelial cell line CCD841 CoN and human colon adenocarcinoma cell line LS180 was examined in response to the investigated *Potentilla* extracts. Cells were exposed to either culture medium (control) or extracts (25–250 μg/ml) for 48 h and, afterward, an MTT test was performed. As presented in Figure 1 and Table 3, all investigated acetone extracts inhibited, in a dose-dependent manner, the metabolic activity of both the normal and cancer colon cell lines. The most significant anticancer effect was achieved by extract **PN7**, which at the highest tested concentration decreased LS180 cell proliferation by 87.3% (IC_50_ PN7 LS180 = 32 μg/ml), while the weakest effect was noted for **PAL7**, which at a concentration of 250 μg/ml inhibited cancer cells division by 57.5% (IC_50_ PAL7 LS180 = 182 μg/ml). In the case of colon epithelial cells, the strongest reduction (49.5%) of their metabolic activity was observed after exposure to 250 μg/ml **PAL7** (IC_50_ PAL7 CCD841 CoN = 233 μg/ml), while the weakest effect, as reflected by the IC_50_ value, was for **PAR7** (IC_50_ PAR7 CCD841 CoN = 1,134 μg/ml). Although all investigated extracts affected both normal and cancer colon cells, LS180 cells were more sensitive to the tested compounds. Comparing the metabolic activity in both analysed cell lines in response to extracts at the corresponding concentrations, greater sensitivity of cancer cells was observed in the entire range of analysed concentrations in the case of **PAR7**, **PRE7**, **PRU7**, and **PN7**, while **PGR7** showed statistically significant differences in concentrations from 50 μg/ml to 250 μg/ml. Even **PAL7** at the highest tested concentrations (150 and 250 μg/ml) strongly inhibited the viability of cancer cells than colon epithelial cells. As a positive control of the experiment, 5-fluorouracil (5-FU) at a concentration of 25 µM was used (Figure 1). The metabolic activity of CCD841 CoN and LS180 cells decreased in response to 5-FU by 22.2% and 46.2%, respectively. Comparing data obtained from the extracts with cell responses to 5-FU revealed that four of six investigated fractions at higher concentrations inhibited the metabolic activity of CCD841 CoN cells more strongly than 25 µM 5-FU: **PAL7** (100, 150, 250 μg/ml); **PRE7** (150, 250 μg/ml); and both **PAR7** and **PRU7** (250 μg/ml). In the case of colon cancer cells, **PGR7**, **PAR7**, **PRE7**, **PRU7**, and **PN7** at concentrations from 50 to 250 μg/ml and **PAL7** at a concentration of 250 μg/ml showed a stronger anti-metabolic effect than the analysed cytostatic.

**TABLE 3:** IC_50_ values (concentration causing viability/proliferation inhibition by 50% compared to control) of acetone extracts isolated from the aerial parts of *Potentilla* L and 5-fluorouracil (5-FU). IC_50_ values were calculated for human colon epithelial cell line CCD841 CoN and human colon adenocarcinoma cell line LS180 based on results of MTT and BrdU assays performed after 48 h of cells treatment with investigated compounds.

Sample	MTT assay						BrdU assay					
	LS180			CCD841 CoN			LS180			CCD841 CoN		
	IC_50_ (µg/ml)	Trust range (µg/ml)	*R* ^2^	IC_50_ (µg/ml)	Trust range (µg/ml)	*R* ^2^	IC_50_ (µg/ml)	Trust range (µg/ml)	*R* ^2^	IC_50_ (µg/ml)	Trust range (µg/ml)	*R* ^2^
**PAL7**	182	169–196	0.983	233	209–261	0.971	12,008	2096–68,805	0.752	4,164	1759–9,859	0.867
**PAR7**	38	32–44	0.974	1,134	575–2,235	0.902	174	165–183	0.982	217	203–231	0.977
**PGR7**	58	50–67	0.957	982	498–1938	0.890	372	338–409	0.968	570	488–666	0.965
**PN7**	32	28–37	0.981	757	459–1,248	0.903	169	159–179	0.974	217	202–233	0.958
**PRE7**	35	30–42	0.969	918	449–1879	0.882	237	223–251	0.966	268	248–289	0.943
**PRU7**	36	30–42	0.974	846	481–1,489	0.916	360	311–416	0.95	538	425–681	0.926
**5-FU**	31	28–33	0.977	113	81–157	0.884	15	13–16	0.956	94	80–111	0.933

**FIGURE 2:**
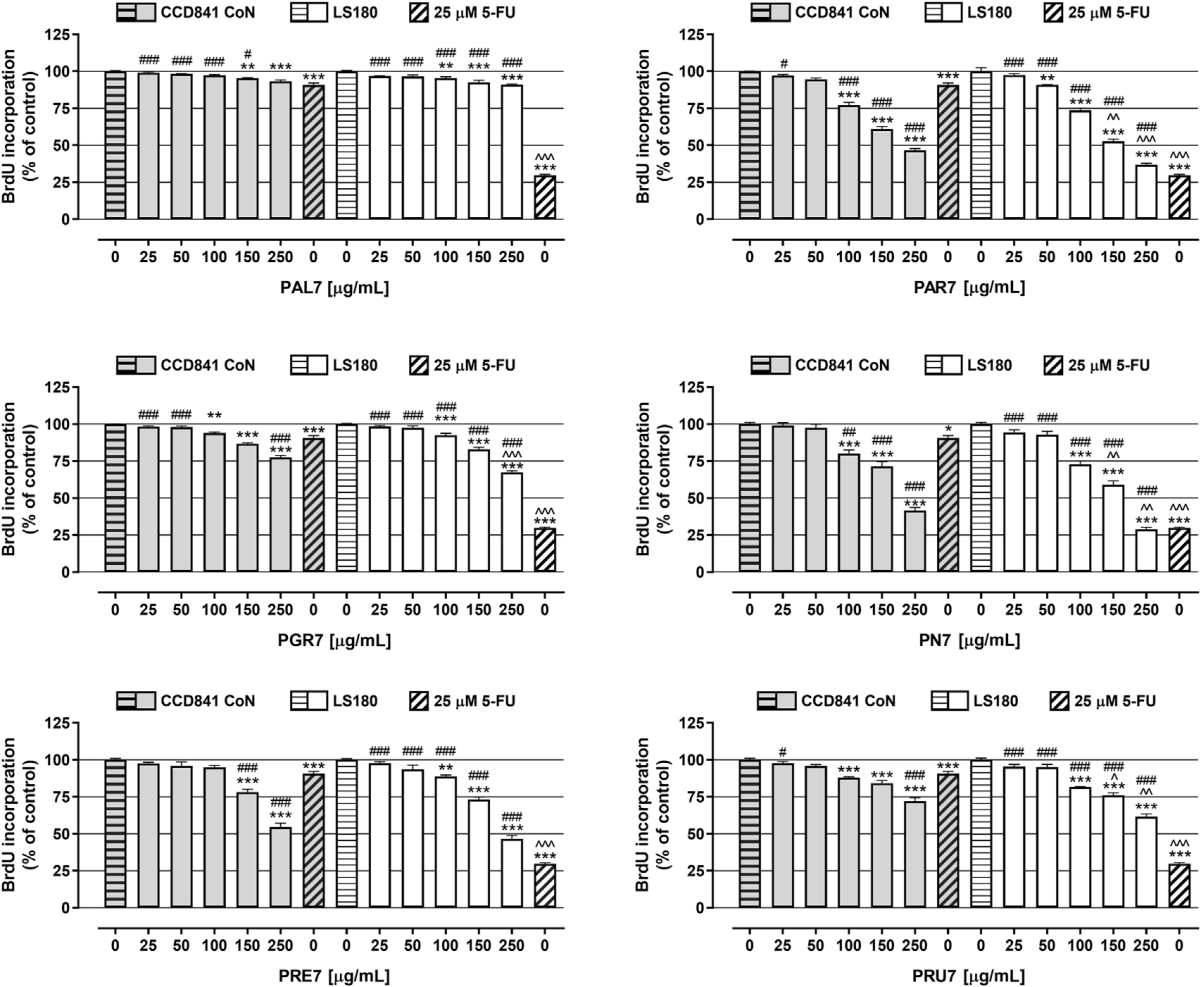
Antiproliferative effect of acetone extracts isolated from the aerial parts of *Potentilla* L. on human colon epithelial cell line CCD841 CoN and human colon adenocarcinoma cell line LS180. The cells were exposed for 48 h to the culture medium alone (control), or the extracts at concentrations of 25, 50, 100, 150, and 250 μg/ml, or 25 μM 5-fluorouracil (5-FU; positive control). The antiproliferative impact of the investigated compounds was measured by immunoassay based on BrdU incorporation into newly synthesized DNA. Results are presented as mean ± SEM of at least six measurements. **p < 0.01; ***p < 0.001 vs. control, #*p* < 0.05; ##*p* < 0.01; ###*p* < 0.001 vs. positive control, ^*p* < 0.05; ^^*p* < 0.01; ^^^*p* < 0.001 colon cancer cells treated with extract/5-FU vs. colon epithelial cells exposed to the extract/5-FU at the corresponding concentration; one-way ANOVA test; post-test: Tukey.

In the next step, the antiproliferative activity of *Potentilla* extracts was assessed in the abovementioned cell lines using BrdU assay (Figure 2; Table 3). A significant decrease of DNA synthesis in colon cancer cells was observed in response to all investigated extracts at concentrations ranging from 100 μg/ml to 250 μg/ml, and simultaneously in the case of **PAR7** a statistically significant antiproliferative effect was also noted at the concentration 50 μg/ml. Furthermore, **PAR7** and **PN7** showed the strongest inhibition of cancer cell proliferation, as reflected by the lowest IC_50_ values (IC_50_ PAR7 LS180 = 174 μg/ml and IC_50_ PN7 LS180 = 169 μg/ml) and the greatest decrease of DNA synthesis in LS180 cells in response to the extracts at a concentration of 250 μg/ml (cell proliferation was reduced by 63.1% (**PAR7**) and 71.1% (**PN7**)). Colon cancer cell division was least inhibited by **PAL7** (IC_50_ PAL7 LS180 = 12 mg/ml), which at the highest tested concentration decreased DNA synthesis by only 9.1%. The investigated extracts also affected the proliferation of colon epithelial cells and statistically significant inhibition of DNA synthesis was noted in response to all compounds at concentrations of 150 and 250 μg/ml, while in the case of **PN7**, **PAR7**, **PRU7**, and **PGR7** the antiproliferative effect was observed also at a concentration of 100 μg/ml. Similar to the colon cancer cells, epithelial cells were the most sensitive to **PN7** and **PAR7**, which at a concentration of 250 μg/ml reduced their proliferation by 58.4% and 53.4%, respectively (IC_50_ PN7 CCD841 CoN = 217 μg/ml and IC_50_ PAR7 CCD841 CoN = 217 μg/ml). The weakest antiproliferative effect in CCD841 CoN cells was observed after exposure to **PAL7**, which at the highest tested concentration inhibited cell division by only 6.7%. Studies have revealed the antiproliferative abilities of *Potentilla* extracts in both normal and cancer colon cells, nevertheless **PGR7** at the highest tested concentration, as well as **PAR7**, **PRU7**, and **PN7** at concentrations 150 and 250 μg/ml, inhibited DNA synthesis significantly more strongly in LS180 cells than CCD841 CoN cells. As presented in Figure 2, 25 µM 5-fluorouracil (5-FU) decreased DNA synthesis in the investigated cell lines by 90.7% (CCD841 CoN) and 29.7% (LS180). The antiproliferative effect of 5-FU observed in colon cancer cells was significantly stronger than changes induced by examined extracts. On the contrary, data collected from colon epithelial cells revealed that five out of six investigated extracts in higher concentrations inhibited DNA synthesis more strongly than 25 µM 5-FU: both **PAR7** and **PN7** (100, 150, 250 μg/ml); **PRE7** (150, 250 μg/ml); and both **PGR7** and **PRU7** (250 μg/ml). The obtained data indicated a higher selectivity of the analysed cytostatic compared with examined extracts in the case of influence on DNA synthesis.

**FIGURE 3:**
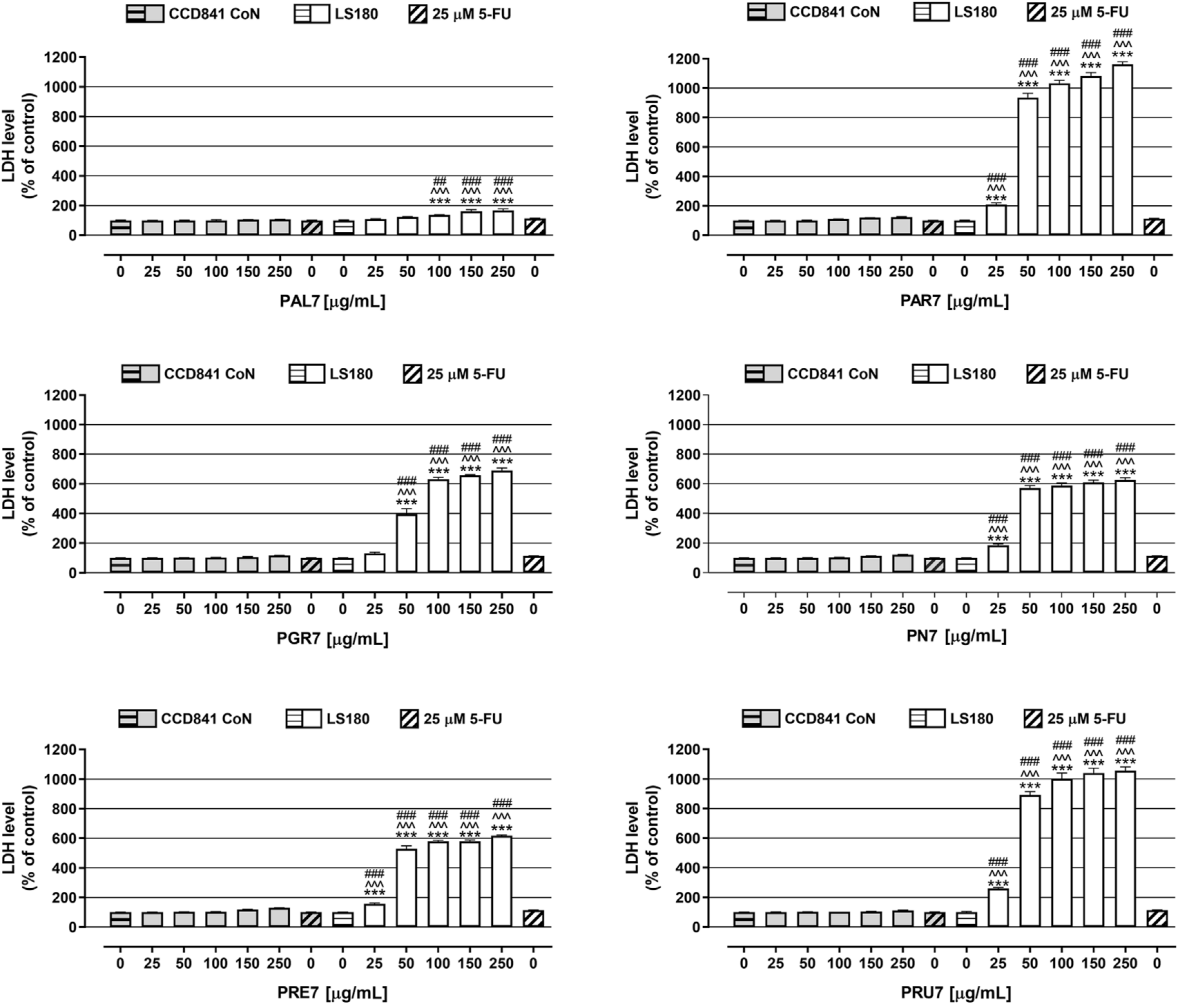
Influence of acetone extracts isolated from the aerial parts of *Potentilla* L. on cell membrane integrity of human colon epithelial CCD841 CoN cells and human colon adenocarcinoma LS180 cells. The cells were exposed for 48 h to the culture medium alone (control), or extracts at concentrations of 25, 50, 100, 150, and 250 μg/ml, or 25 μM 5-fluorouracil (5-FU; positive control). Compound cytotoxicity (level of LDH released into the cell culture medium from damaged cell membranes) was measured using LDH assay. Results are presented as mean ± SEM of at least six measurements. ***p < 0.001 vs. control, #*p* < 0.05; ##*p* < 0.01; ###*p* < 0.001 vs. positive control, ^^^*p* < 0.001 colon cancer cells treated with extract/5-FU vs. colon epithelial cells exposed to the extract/5-FU at the corresponding concentration; one-way ANOVA test; post-test: Tukey.

In the last step of the *in vitro* studies, extracts cytotoxicity was examined in CCD841 CoN cells and LS180 cells using LDH-based assay. As presented in Figure 3, the tested extracts were not cytotoxic against human colon epithelial cells, while they significantly damaged the cell membranes of colon cancer cells, and the observed effect was dose-dependent. The strongest release of LDH was noted in LS180 cells in response to **PAR7**, which in concentrations ranging from 25 to 250 μg/ml increased the LDH level by 110% and 1,062%, respectively. Very similar results were obtained after LS180 cell exposure to **PRU7**, which in the mentioned range of concentrations increased LDH release by 161% (25 μg/ml) and 956% (250 μg/ml). The weakest cytotoxic effect was noted in colon cancer cells treated with **PAL7**, which at the highest tested concentration caused an increase in the LDH level of 68%. Used as a positive control, 5-FU at a concentration of 25 µM was not cytotoxic against colon epithelial or colon cancer cells (Figure 3). The LDH levels of the cells were 100.7% (CCD841 CoN) and 113.4% (LS180). All investigated extracts damaged colon cancer cell membranes more effectively than 5-FU, and this difference was especially evident in the case of **PRU7**, **PAR7**, **PN7**, and **PRE7**, which even at the lowest tested concentration (25 μg/ml) increased the LDH level to 261, 210, 185, and 156%, respectively.

## Discussion

Many studies have shown that *Potentilla* species are a source of a wide spectrum of secondary metabolites, mainly polyphenols, such as hydrolysable and condensed tannins, flavonoids and their glycosides, and phenolic acids, which show a variety of biological activities (Augustynowicz et al., 2021b). *Potentilla* species have a long history of use to treat intestinal problems, such as diarrhoea, inflammatory bowel disease (Tomczyk and Latte, 2009). Selected species for the present study are common in the Europe and were rarely selected as a subject of anticancer evaluation. Basing on the literature search we hypothesized that similarly to other species from this genus would exhibit anticancer potential. The quantitative identification of polyphenolic classes present in extracts using colorimetric methods offers information on their general contents. In our study, high TPC, TFC, and TTC values were observed in all tested samples. The TPC and TFC results were significantly higher compared to previous studies on aerial parts of *P. argentea, P. grandiflora, P. recta,* and *P. norvegica* (Tomczyk et al., 2010; Sut et al., 2019; Augustynowicz et al., 2021a). The differences in results can be partially explained by the type of solvent used in the extraction process. Aqueous acetone during the extraction process inhibits the interaction between tannins and proteins, and decreases the cleaving of depside bonds in hydrolysable tannins in comparison to aqueous alcohols. These mechanisms may lead to higher contents of high-molecular tannins in acetone extracts (Hagerman, 1988; Mueller-Harvey, 2001). Moreover, on several occasions, acetone has been reported as a good solvent for the extraction of flavonoids with higher contents, in comparison to water and alcoholic solvents (Dirar et al., 2019; Patel and Ghane, 2021). Furthermore, LC-PDA-HRMS analysis revealed a number of polyphenols, such as ellagitannins and products of their degradation, flavonoids, and phenolic acids, that were present in all extracts. Ellagitannins are plant secondary metabolites that tend to form relatively high molecular weight dimers and oligomers, ranging from 300 to 20,000 Da. Plants from the Rosaceae family accumulate a series of oligomeric, macrocyclic oligomeric, and *C*-glycosidic ellagitannins that can be used as chemophenetic markers (Grochowski et al., 2017; Gesek et al., 2021). On several occasions the presence of dimeric ellagitannin - agrimoniin in aerial parts of *Potentilla* species, in particular *P. anserina and P. kleiniana, P. recta*, have been reported (Okuda et al., 1982; Fecka, 2009; Bazylko et al., 2013). The chromatographic analysis reported herein indicates that agrimoniin is the most abundant ellagitannin in all extracts except **PAL7**. Moreover, several other phenolic compounds, such as pedunculagin, laevigatins, brevifolincarboxylic acid, and ellagic acid, an artifact, are released as a product of hydrolysis of ellagitannins. These compounds are widely present in the aerial parts of different species belonging to the genus *Potentilla*, including *P. indica, P. freyniana, Duchesnea chrysantha,* and *P. anserina*, and therefore could be considered significant in the chemophenetics of this genus (Okuda et al., 1992; Lee and Yang, 1994; Fecka, 2009; Luo et al., 2020). Furthermore, flavonol derivatives, such as quercetin 3-*O*-rutoside, quercetin 3-*O*-galactoside, quercetin 3-*O*-glucuronide, quercetin 3-*O*-arabinoside, kaempferol 3-*O*-glucoside, kaempferol 3-*O*-glucuronide, and tiliroside, were found in at least one of the 17 investigated *Potentilla* species (Tomczyk and Latte, 2009; Augustynowicz et al., 2021b). More interestingly, *N*-acylated biogenic amine derivative, N1, N5, N10-tricoumaroyl spermidine, was reported for the first time in the genus *Potentilla*. This compound accumulates exclusively in the pollen coat and has been detected in several other genera in the Rosaceae family (Elejalde-Palmett et al., 2015).

The anticancer potential of acetone extracts isolated from selected *Potentilla* species was examined in both colon cancer LS180 cells as well as normal colon epithelial CCD841 CoN cells by investigation compounds influence on cell viability (MTT assay), proliferation (BrdU assay), and cytotoxicity (LDH assay). All investigated extracts decreased viability of both normal and cancer colon cells in a dose-dependent manner; however, LS180 cells were more sensitive to the tested compounds. The results of MTT assay indicated that the tested extracts effectively decreased the mitochondrial metabolism of human colon cancer cells, which could be associated with the presence of hydrolysable tannins in all extracts except the **PAL7**, which revealed the weakest anticancer effect. Moreover, the highest impact in decrease of cancer cells viability by **PAR7**, **PN7** and **PRU7** correlate with their highest TPC and TTC values. Agrimoniin was shown to have prominent antioxidative, anti-inflammatory, and anticancer effects. Hoffman et al. (2016) found that agrimoniin-enriched fractions from rhizomes of *P. erecta* directly inhibit UVB-induced cyclooxygenase-2 (COX-2) expression and production of PGE2 in human keratinocytes (HaCaT), as well as in an *in vivo* model, and inhibit epidermal growth factor receptor (EGFR) phosphorylation. Shi et al. (2015) demonstrated that lyophilized strawberries (*Fragaria* x *ananasa*, Rosaceae) containing 16.2% agrimoniin downregulated the mRNA expression of COX-2, IL-1β, IL-6, TNF-α, and iNOS in AOM/DSS-induced colon cancer in mice. BrdU assay revealed a significant decrease of DNA synthesis in both colon cancer and non-cancer cells in response to all investigated extracts. The strongest antiproliferation effect in cancer cells was observed after treatment with **PAR7** and **PN7**. Those extracts revealed to posess the highest total polyphenol and tannin contents. Notably, the antiproliferative effect of 5-FU observed in colon cancer cells was significantly stronger than that of the examined extracts. Similarly, data collected from colon epithelial cells revealed that five out of six investigated extracts in higher concentrations inhibited DNA synthesis stronger than the positive control. The *in vivo* bioavailability of high weight ellagitannins is relatively low. Ellagitannins at neutral or alkaline pH are hydrolysed with the release of free ellagic acid, which exerts a number of biological activities (Ismail et al., 2016). Whitley et al. (2003) found that the human colorectal adenocarcinoma cell line Caco-2 strongly accumulated ellagic acid and, furthermore, 93% of it was irreversibly bounded to cellular DNA and proteins. Moreover, ellagic acid significantly decreased the expression of genes involved in the p53, PI3K-Akt, mitogen-activated protein kinase (MAPK), and TGF-β signaling pathways in human colorectal carcinoma cell line HCT 116 (Zhao et al., 2017). Ellagic acid also reduced the viability of human nasopharyngeal carcinoma cell line NPC-BM1 via activation of caspase-3 and inhibition of Bcl-2 and telomerase (Huang et al., 2009). Our results are in agreement with the studies by Kowalik and co-authors (2020), showing that selected extracts and fractions from aerial parts of *P. alba* significantly decreased proliferation of human colon cancer HT-29 cells. Additionally the authors found out that selected extracts and fractions from *P. alba* increased proliferation of human normal epithelial CCD 841 CoTr cells. Moreover, the tested samples damaged cell membranes and decreased their viability (Kowalik et al., 2020). Kaempferol 3-*O*-glucoside, present in all investigated extracts, exhibits anti-inflammatory, antioxidant, and anticancer effects. A recent study conducted on human colon cancer HCT 116 cells revealed that kaempferol 3-*O*-glucoside induces cell apoptosis by increasing expression of pro-apoptotic caspases (caspase 3, caspase 6, caspase 7, caspase 8, and caspase 9), protein p53, and Bax, and decreasing expression of anti-apoptotic proteins, cleaved caspase 3, and Bcl-2. Moreover, the investigated compound causes G0/G1 arrest, inhibits the expression of metalloproteinases MMP-2 and MMP-9, and decreases the activity of the NF-κB signalling pathway (Yang et al., 2021). Notably, tiliroside isolated from *P. argentea* exerted inhibitory activity against topoisomerase I and II and showed moderate cytotoxicity against human breast carcinoma cell line MCF-7 (Tomczyk et al., 2008). Finally, the LDH assay showed that the tested extracts even at the lowest concentration (25 μg/ml) significantly damaged the cell membranes of investigated colon cancer cells, releasing the high doses of LDH into the cell culture medium. The weakest effect was observed for PAL7, which may be due to the absence of hydrolysable tannins, which modify the permeability of cell membranes. However, strong observed effect of rest of tested extracts can be explained by high TTC. Moreover, the exerted the strongest cytotoxic effects of **PAR7** and **PRU7** among all extracts can be explained by their higher TPC and TTC values. At the same time, all tested samples were not cytotoxic against normal colon cells. In a recent paper, Borisowa and co-authors (2019) found that hydrolysable tannins selectively block calcium-activated chloride channels and form selective pores in the cell membrane (Borisova et al., 2019). Moreover, pedunculagin increased cytotoxicity of 5-FU against human liver cancer cells QGY-7703, probably through increased permeability of the cancer cell membrane, as observed by the authors through a microscope (Xiao et al., 2012). A recent study revealed that agrimoniin stimulates apoptosis via the mitochondria pathway, inducing activity of the mitochondrial permeability transition pore (MPTP), which leads to mitochondria swelling and a decrease in energy production. Moreover, the authors found that agrimoniin is cytotoxic against K562 and HeLa cell lines (Fedotcheva et al., 2021). The *in vivo* effects of tannin-rich acetone extracts from selected *Potentilla* species may vary from obtained *in vitro* results. A recent study on aerial parts of *P. anserina* and rhizomes of *P. erecta* revealed that human intestinal microbiota convert ellagitannins to urolithins, which possess potent anti-inflammatory and anticancer activities (Piwowarski et al., 2014). Moreover, several studies suggest that the chronic application of tannin-rich extracts may lead to iron-deficiency anemia. Hydrolysable tannins posess antinutritional properties, due to their potential to complex iron ions and reduce their absorption (Petry et al., 2010). However, those effect may be offset by the development of formulations with modified release of extract or by the inclusion in diet of other bioactives, such as ascorbic acid, which prevents the inhibitory effect of polyphenols on iron absorption (Petroski and Minich, 2020). The acute complications of advanced stages of colorectal cancers includes a number of complications, such as bleeding, perforation and/or obstruction (Yang and Pan, 2014). Hydrolysable tannins are well known for their anti-bleeding properties. The tannin-rich extracts from *Potentilla* species may be used as potent, plant-based styptic agents as a complementary therapy in advanced stages of colorectal cancers.

## Conclusion

In conclusion, this study reports, for the first time, analysis of the LC-PDA-HRMS profile of acetone extracts of selected *Potentilla* species. The analysis revealed the presence of several phenolic compounds, such as agrimoniin, pedunculagin, brevifolincarboxylic acid, ellagic acid, tiliroside, and tricoumaroyl spermidine. These secondary metabolites can be considered as chemophenetic markers for the genus *Potentilla*. Four of six investigated extracts (**PAR7**, **PRE7**, **PRU7**, **PN7**) showed great chemopreventive potential, manifested by the effective elimination of colon cancer cells, causing both damage to their cell membranes and inhibition of their proliferation and metabolic activity, with a simultaneous lack of a cytotoxic effect on normal colon epithelial cells and a significantly weaker effect on their metabolism and DNA synthesis compared to cancer cells. While it is impossible to specify the extract with the greatest therapeutic potential, these studies unequivocally showed that **PAL7** had the lowest anti-cancer potential in a cellular model of colon cancer.

## Data Availability

The original contributions presented in the study are included in the article/Supplementary Material, further inquiries can be directed to the corresponding author.
