# Supplementary material for: Anticancer potential of acetone extracts from selected Potentilla species against human colorectal cancer cells
Source: Front Pharmacol. 2022 Sep 29;13:1027315. doi: 10.3389/fphar.2022.1027315 (PMC9556846; doi:10.3389/fphar.2022.1027315)
Supplement: Supplementary file 1 [file DataSheet1.docx]

Supplementary Material


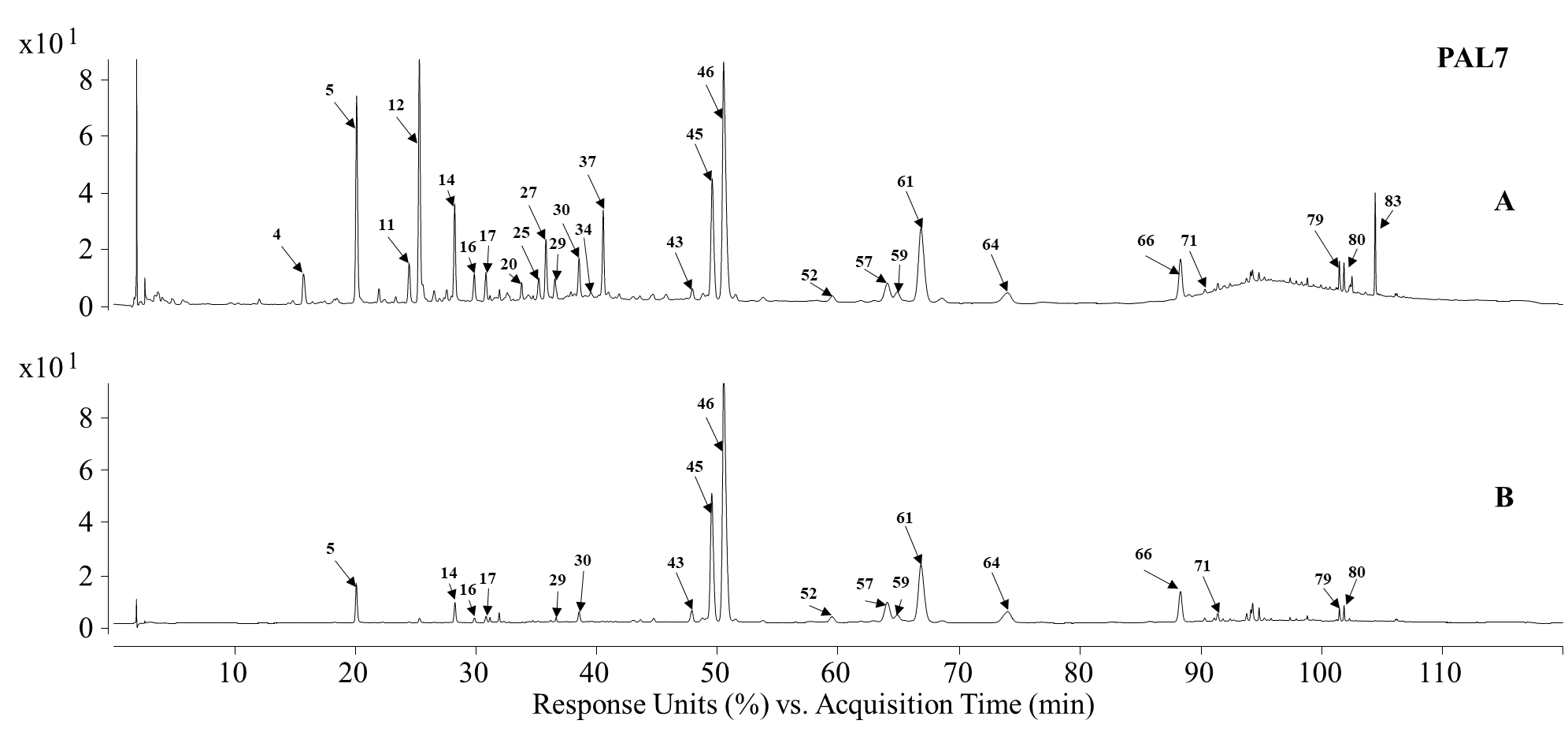


**Supplementary Figure 1.** The UV chromatograms with a designation of the main components of the analyzed PAL7 extract, recorded at lengths of 280 nm (A) and 360 nm (B).


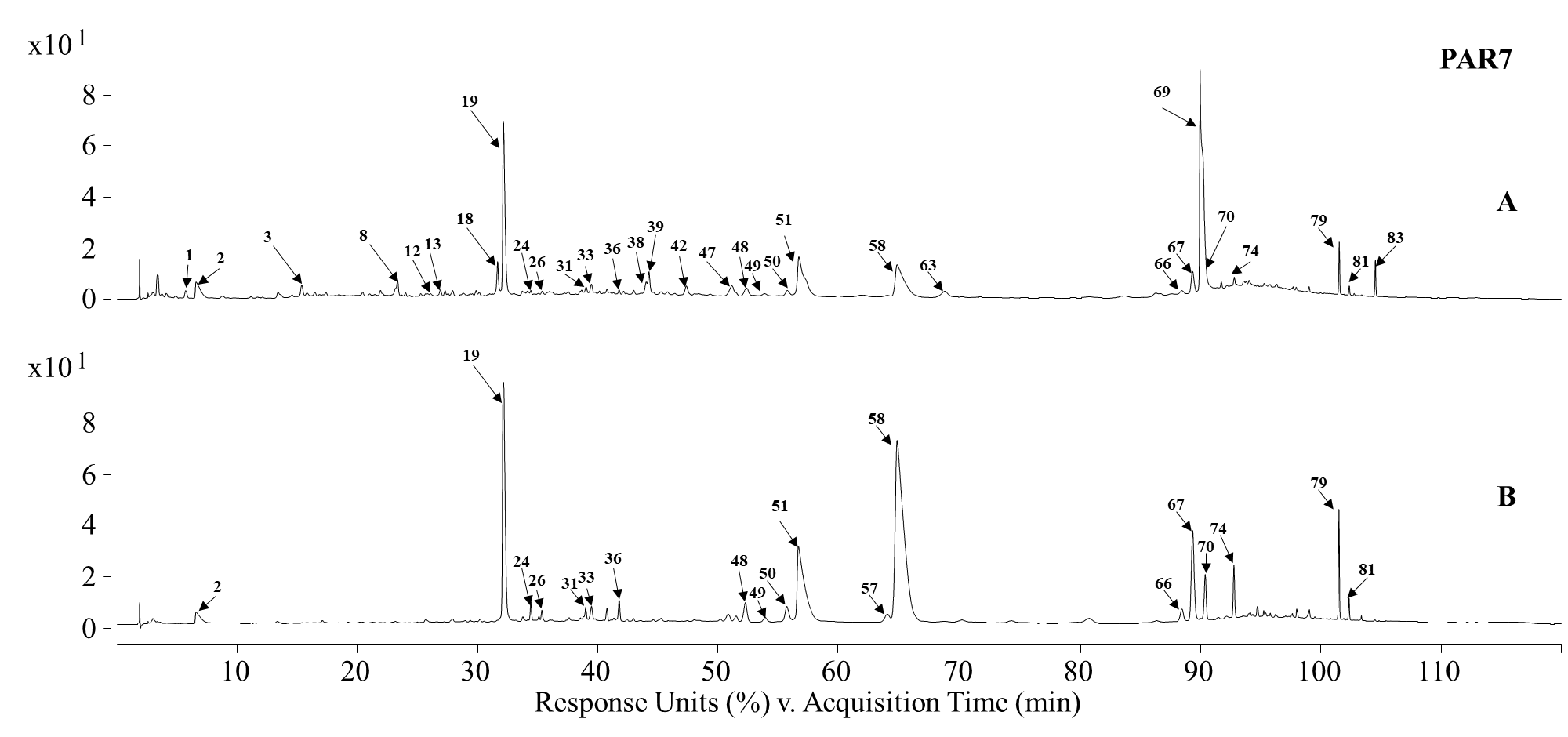


**Supplementary Figure 2.** The UV chromatograms with a designation of the main components of the analyzed PAR7 extract, recorded at lengths of 280 nm (A) and 360 nm (B).


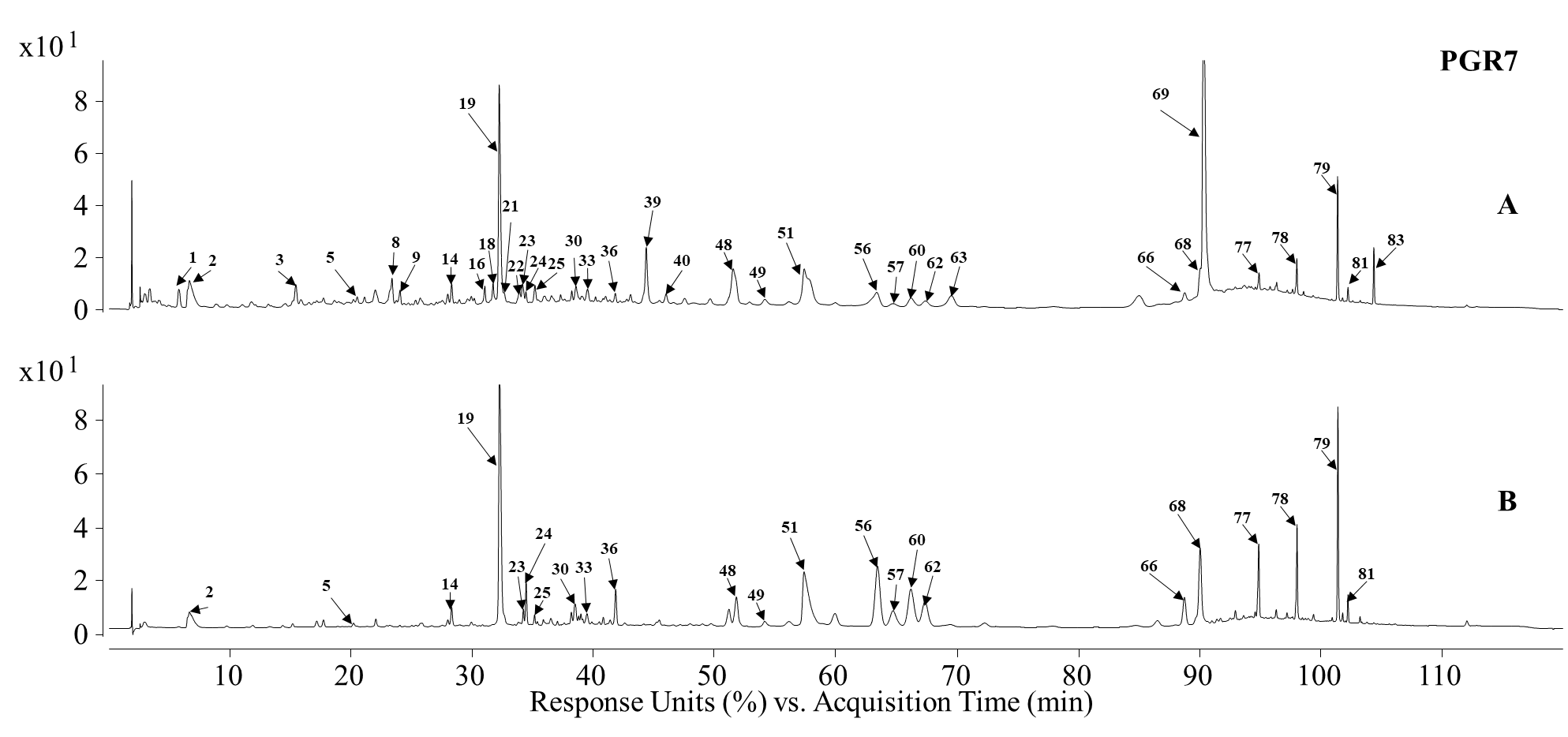


**Supplementary Figure 3.** The UV chromatograms with a designation of the main components of the analyzed PGR7 extract, recorded at lengths of 280 nm (A) and 360 nm (B).


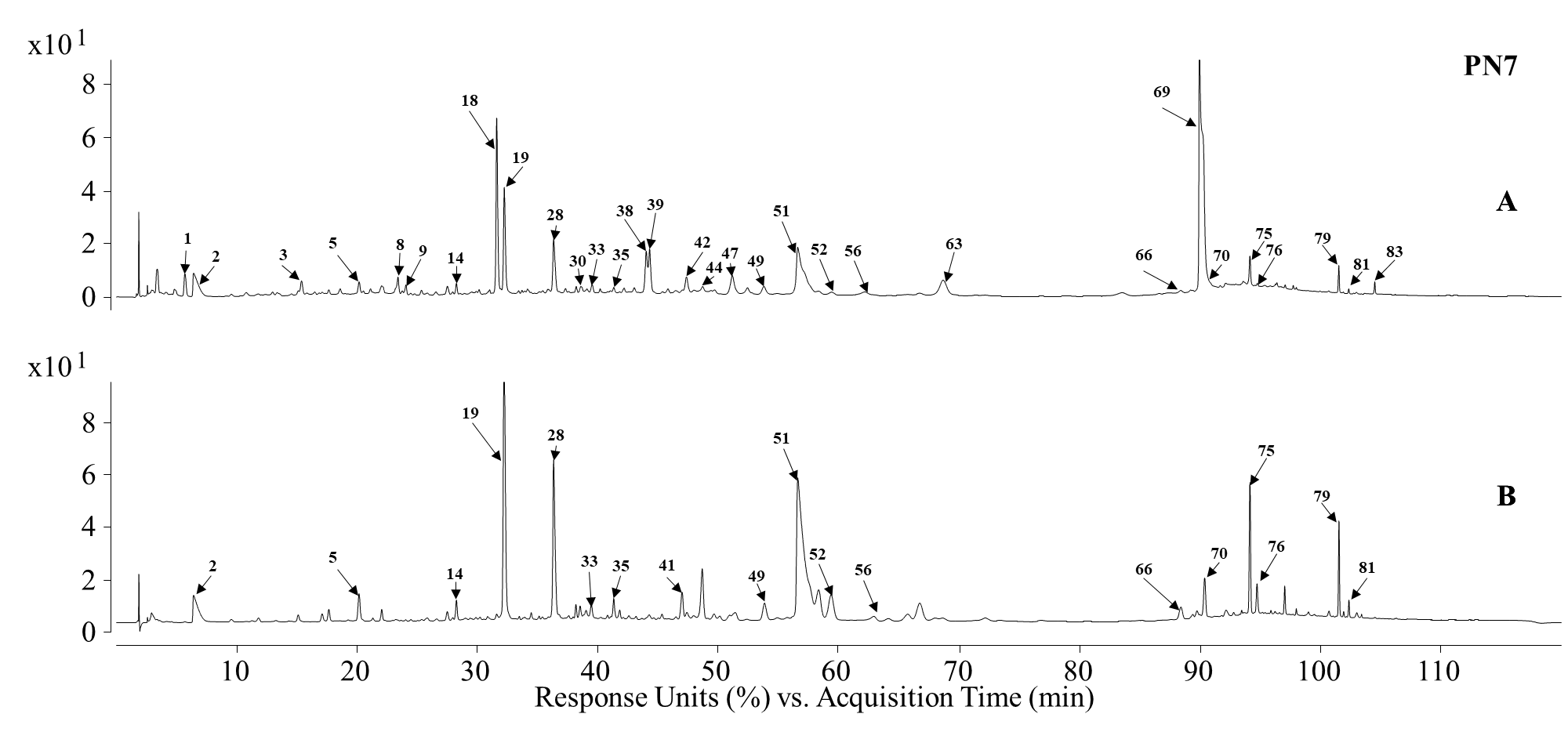


**Supplementary Figure 4.** The UV chromatograms with a designation of the main components of the analyzed PN7 extract, recorded at lengths of 280 nm (A) and 360 nm (B).


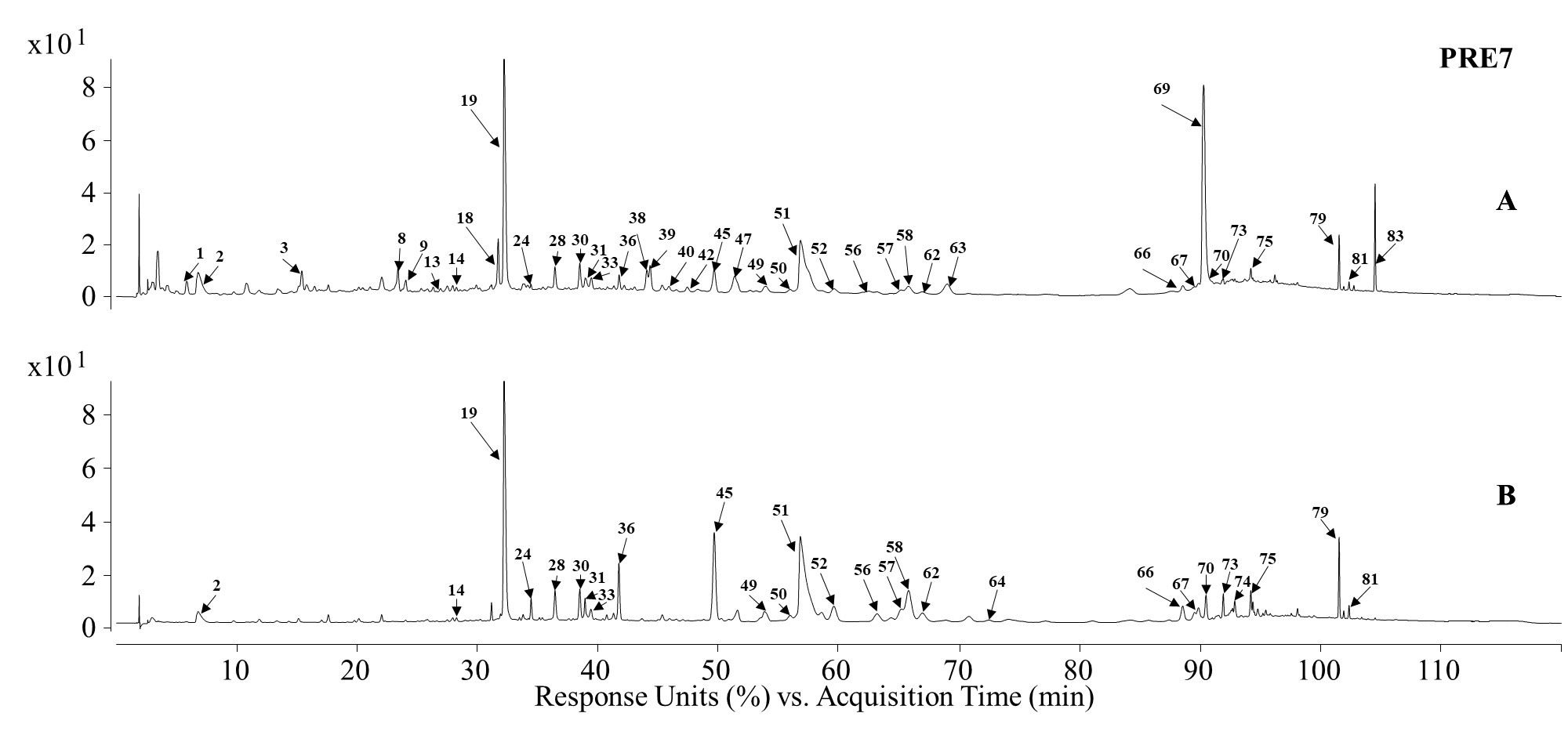


**Supplementary Figure 5.** The UV chromatograms with a designation of the main components of the analyzed PRE7 extract, recorded at lengths of 280 nm (A) and 360 nm (B).


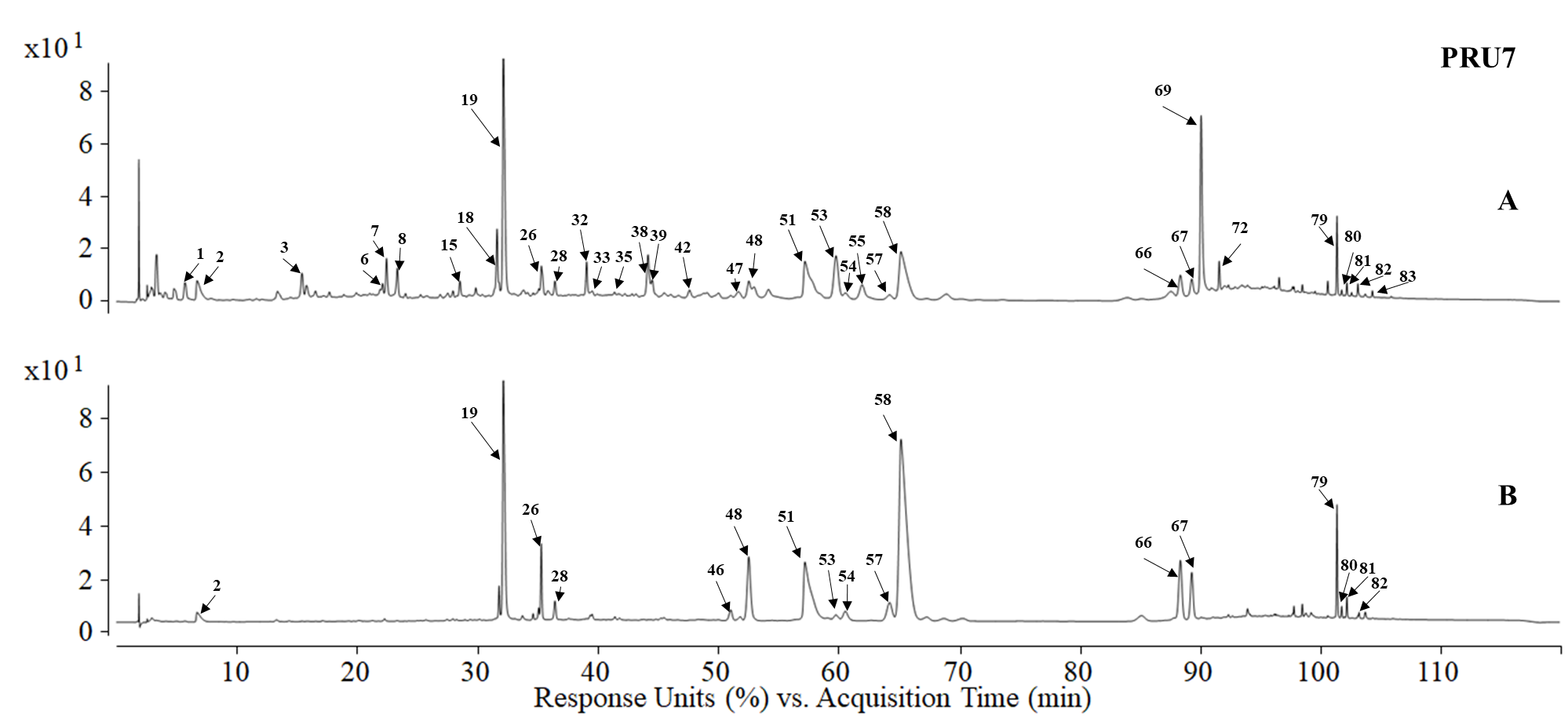


**Supplementary Figure 6.** The UV chromatograms with a designation of the main components of the analyzed PRU7 extract, recorded at lengths of 280 nm (A) and 360 nm (B).
